# Diagnosis of chronic allograft vasculopathy using semiquantitative stress perfusion CMR in heart transplant patients

**DOI:** 10.1186/1532-429X-18-S1-Q13

**Published:** 2016-01-27

**Authors:** Madeline Schwid, Hannah Recht, Jeremy D Collins, Michael Markl, Daniel C Lee, James C Carr

**Affiliations:** grid.465264.7Northwestern University, Chicago, IL USA

## Background

Patients with heart transplant are susceptible to developing chronic allograft vasculopathy (CAV) which increases in frequency over time. Traditional methods for diagnosing CAV rely on invasive catheter based angiography and intravascular ultrasound (IVUS). Stress perfusion cardiac MRI (CMR) is frequently used to noninvasively diagnose ischemic heart disease and has also been used to assess CAV in heart transplant patients. The purpose of this study was to evaluate semiquantitative measures of ischemia using noninvasive stress perfusion CMR in the detection of CAV using invasive coronary angiography and IVUS as the standard reference.

## Methods

A cohort of 43 chronic heart transplant patients was studied using both stress CMR and coronary angiography. Stress CMR was performed in the standard manner using an IV injection of regadenoson and 0.2 mM/kg of a gadolinium based contrast agent. Based on the coronary angiogram, the patients were divided into groups of no disease (group 1), mild chronic allograft vasculopathy (CAV) (group 2), and moderate to severe CAV (group 3), based on the International Heart Lung Transplantation (IHLT) guidelines for diagnosis of CAV. The time between the angiogram and perfusion results varies from less than 6 months to just over 2 years, with an average of about 1 year between studies. The rest and stress perfusion images were analyzed by segment based on the AHA 16 segment model of the heart using dedicated myocardial perfusion software (Argus, Siemens Healthcare). The upslope was measured for each segment on both stress and rest images and then an average upslope value was calculated for each patient. The myocardial perfusion reserve was calculated by dividing the upslope values of rest and stress images. The semiquantitative results of the perfusion studies, both stress perfusion upslope values and myocardial perfusion reserve, were then correlated to the angiogram results.

## Results

Group 1 consisted of 19 patients, group 2 had 19 patients, and group 3 had 5 patients. The calculated upslopes for each group were as follows: 3.28, 3.03, and 2.30, for groups 1, 2, and 3, respectively. There was a statistical difference between group 1 and 3 (p = 0.035), and no statistical difference between groups 1 and 2 (p = 0.234), and groups 2 and 3 (p = 0.054). For myocardial perfusion reserve, the calculated ratios were 4.71, 3.66, and 2.36 for groups 1, 2, and 3, respectively. The difference between groups 1 and 3 was significant (p = 0.033), while the differences between groups 1 and 2 (p = 0.176), and groups 2 and 3 (p = 0.073) were not.

## Conclusions

Semiquantitative parameters of upslope and myocardial perfusion reserve as measured from stress perfusion CMR can detect moderate to severe CAV in heart transplant patients. Stress perfusion CMR is a promising noninvasive tool for detecting CAV potentially obviating the need for regular invasive coronary angiography.

**Figure 1:**
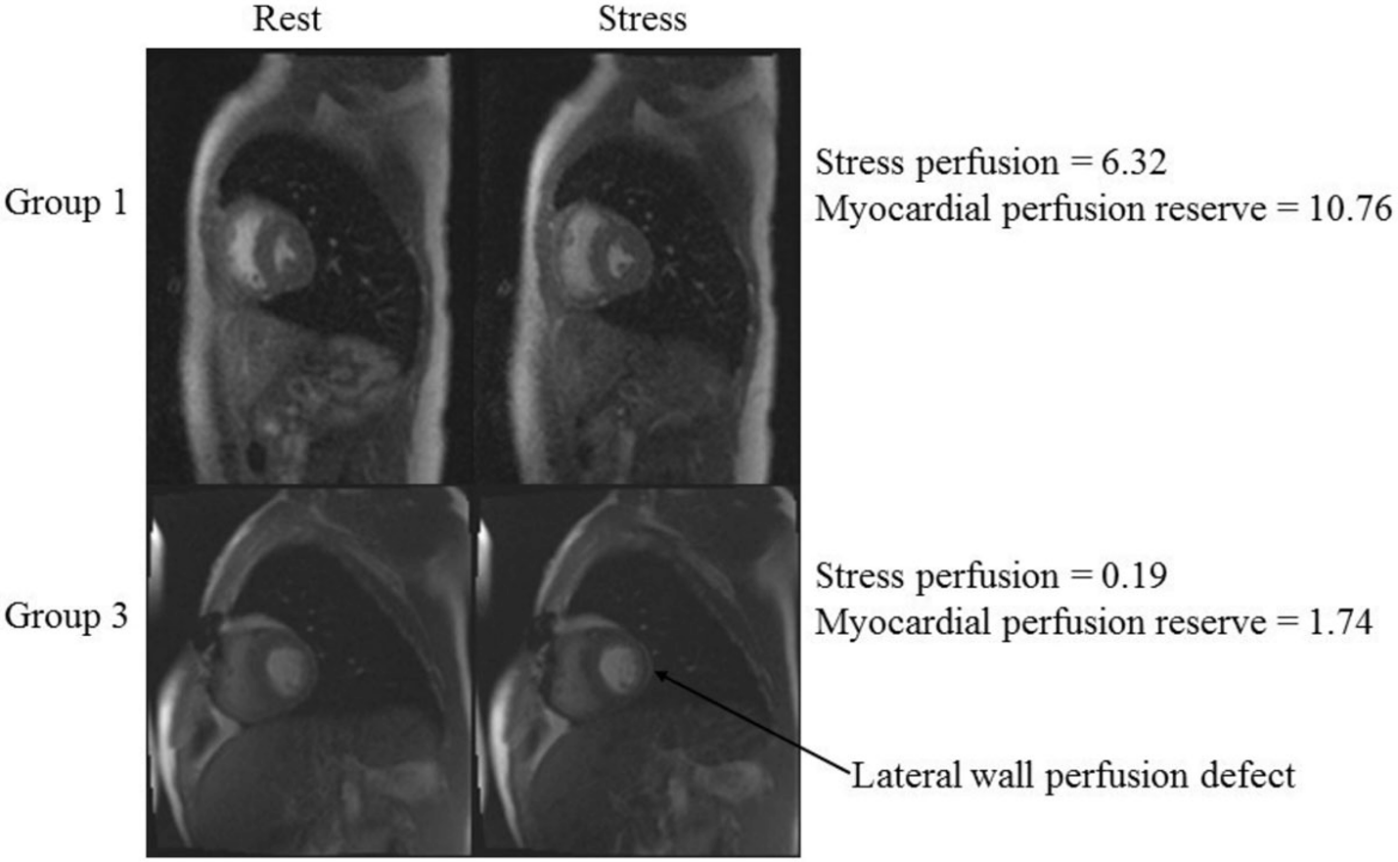
**This image demonstrates the difference between cardiac perfusion at rest and stress between a patient without disease (group 1) and a patient with CAV (group 3)**. The stress image for the group 3 patient shows a perfusion defect in the lateral wall and decreased upslope and myocardial perfusion reserve as compared to the group 1 patient.

